# Immunohistochemical Localization of the Bradykinin B1 and B2 Receptors in Human Nasal Mucosa

**DOI:** 10.1155/2009/102406

**Published:** 2009-04-23

**Authors:** Hideaki Shirasaki, Etsuko Kanaizumi, Tetsuo Himi

**Affiliations:** Department of Otolaryngology, School of Medicine, Sapporo Medical University, S 1 W 16, Chu-ku Sapporo, 060-8543, Japan

## Abstract

Bradykinin (BK) has been tobe thought a potent mediator involved in allergic rhinitis because BK was recovered from the nasal lavage fluid of allergic rhinitis patients after allergen provocation and BK receptor antagonists relief nasal allergic symptoms. Two mammalian BK receptor subtypes, B1 and B2, have been defined based on their pharmacological properties. We investigated the localization of these receptors by immunohistochemistry. Human turbinates were obtained after turbinectomy from 12 patients with nasal obstruction refractory to medical therapy. The immunohistochemical study revealed that epithelial cells, submucosal glands, fibroblast, vascular smooth muscle, vascular endothelial cells, and macrophages showed immunoreactivity for both B1 and B2 receptors. The B2 receptor expression was found in peripheral nerve fibers, whereas the B1 expression was not observed in nerves. The results may have an important clinical implication for understanding the differential roles of BK receptor subtypes on upper airway diseases such as allergic rhinitis and nonallergic rhinitis.

## 1. Introduction

The allergic response is a complex process involving the interaction of many mediators. Bradykinin (BK) is a potent inflammatory mediator and its actions are mediated via specific cell surface receptors which are coupled to G-proteins. Two mammalian BK receptor subtypes, B1 and B2, have been reported, and the amino acid sequence of the B1 receptor is 36% identical to the amino acid sequence of the B2 receptor [1]. Administration of exogenous BK into human nasal airway causes nasal obstruction, rhinorrhea, and nasal pain [2, 3]. These effects appear to be mediated by bradykinin B2 receptor because bradykinin B2 receptor antagonists abolish bradykinin-induced nasal obstruction and plasma extravasation, whereas agonists at the bradykinin B1 receptor do not cause any symptoms [3]. Icatibant, a bradykinin B2 receptor antagonist, inhibits the immediate inflammatory response to antigen in subjects with perennial allergic rhinitis [4, 5]. These reports suggest that BK may play an important role in the pathogenesis of allergic rhinitis. The previous autoradiografic study using ^125^I-BK has demonstrated specific ^125^I-BK binding sites mainly exist on the small muscular arteries, venous sinusoids, and submucosal fibers in human nasal mucosa [6]. However, there has been no other report about BK receptor expression in upper airway. 

In the present study, immunohistochemistry for bradykinin B1 and B2 receptors was performed to confirm the expression and the distribution of these receptors in human nasal mucosa. 

## 2. Materials and Methods

### 2.1. Tissue Preparation

Human inferior turbinates were obtained after turbinectomy from 12 patients with nasal obstruction refractory to medical therapy. Informed consent was obtained from all patients and this study was approved by the ethics committee of Sapporo Medical University. All were nonsmokers, and 6 patients had perennial allergy against mites as defined by questionnaire and CAP test (Pharmacia, Uppsala, Sweden). All medications, including antibiotics, were prohibited for at least 3 weeks prior to the study. Demographic and clinical characteristics of the patients are summarized in Table 1. The nasal mucosal specimens were immediately fixed in 10% formalin for immunohistochemistry. 

### 2.2. Immunohistochemistry

#### 2.2.1. Antibodies

For immunohistochemistry of B1 receptor, rabbit antihuman B1 receptor polyclonal antibody against a peptide corresponding to C-terminal domain of human B1 receptor (catalog # LS-A3580, Lifespan Biosciences, Mich USA) was used at 1:20 dilutions. Similarly, for immunohistochemistry of B2 receptor, rabbit antihuman B2 receptor polyclonal antibody against a peptide corresponding to C-terminal domain of human B2 receptor (catalog *#* LS-A797, Lifespan Biosciences, Mich, USA) was used at 1:100 dilutions. To identify the subsets of cells expressing each bradykinin receptor, the following monoclonal antibodies were used: anti-CD68 (KP-1 clone, Dako Corporation, Carpinteria, Calif, USA) for macrophage, anti-CD31 (JC70A clone, Dako) for vascular endothelial cells, antihuman fibroblast (5B5 clone, DAKO) for fibroblast, anticytokeratin (AE1/AE3 clone, Dako) for epithelial cells, and antineurofilament protein (2F11 clone, Dako) for peripheral nerves. 

#### 2.2.2. Immunohistochemistry

Deparaffinized sections were initially incubated with 3% H_2_O_2_ in methanol for 10 minutes to quench endogenous peroxidase activity. After microwave treatment (10 minutes at 500 Watt in citrate buffer), the sections were incubated in blocking solution (10% normal goat serum in PBS) for 30 minutes before incubation in primary antibody. Then, the sections were incubated with anti-bradykinin B1 or B2 polyclonal antibody for overnight at 4°C, washed, and incubated for 30 minutes with EnVision+, Peroxidase (Dako). A further washing in PBS was followed by developing in DAB (Dako) as a chromogen for signal visualization. The slides were counterstained Mayer's haematoxylin and coverslipped using mounting medium. 

To identify the subsets of cells expressing each bradykinin receptor, some sections were stained by immunofluorescence technique. For double staining, deparaffinized sections were incubated overnight at 4°C with a combination of rabbit polyclonal antihuman bradykinin B1 or B2 antibody and one of mouse monoclonal antihuman phenotypical makers antibody. Sections were washed in PBS and were incubated for 30 minutes with Alexa Fluor 594-labelled goat antimouse IgG (diluted 1:50; Molecular Probes, Ore, USA) and Alexa Fluor 488-labelled goat antirabbit IgG (diluted 1:50; Molecular Probes). Sections were mounted with SlowFade antifade kits (Molecular Probes) and examined under Olympus BX51 microscope, DP70 CCD camera (Olympus Optical Co., Tokyo, Japan). All images were processed with DP Controller and DP Manager software (Olympus Optical Co) for image analysis. Using this method, bradykinin B1 or B2 receptor expressing cells was green, cellular phenotypical makers were red, and the combined signal is visualized as yellow. Negative controls were obtained by replacing primary antibodies by mouse IgG1 and rabbit immunoglobulin fraction (Dako). 

## 3. Results

**Figure 1:**
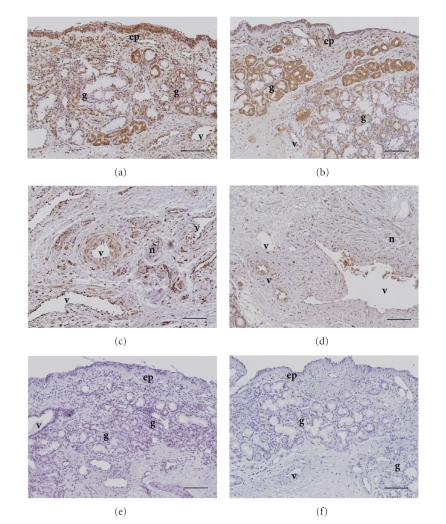
Immunohistochemical staining for bradykinin B1 receptor in human allergic (a), (c), (e) and nonallergic (b), (d), (f) nasal mucosa. Inferior turbinates were stained with antihuman B1 receptor antibody (a)–(d) or normal rabbit immunoglobulin (e), (f). Immunoreactivity for B1 receptor was significantly detected in submucosal glands (a), (b), epithelial cells (a), (b), and fibroblasts (c), (d). ep: epithelial cells; v: blood vessels; g: submucosal glands; n: nerves. Scale bar = 100 *μ*m.

**Figure 2:**
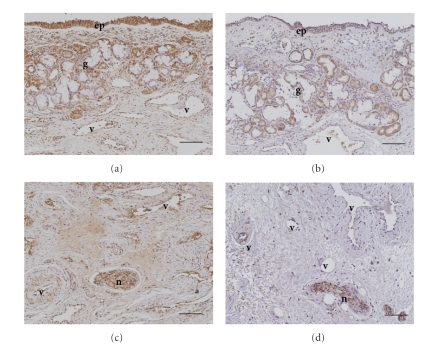
Immunohistochemical staining for bradykinin B2 receptor in human allergic (a), (c), (e) and nonallergic (b), (d), (f) nasal mucosa. Inferior turbinates were stained with antihuman B2 receptor antibody (a)–(d) or normal rabbit immunoglobulin (e), (f). Immunoreactivity for B2 receptor was significantly detected in submucosal glands (), epithelial cells (c), vascular smooth muscle (c), (d), nerve bundle, and fibroblasts (d). ep: epithelial cells; v: blood vessels; g: submucosal glands; n: nerves. Scale bar = 100 *μ*m.

As shown in Figure 1, the immunoreactivity for B1 receptor was significantly detected in submucosal glands, epithelial cells (Figures 1(a), 1(b)), and fibroblasts (Figures 1(c), 1(d)). Immunoreactivity for B2 receptor was significantly detected in submucosal glands, epithelial cells (Figures 2(a), 2(b)), vascular smooth muscle (Figures 2(c), 2(d)), nerve bundle, and fibroblasts (Figures 2(c), 2(d)). Specificity of the staining was also confirmed by the absence of labeling with normal rabbit immunoglobulin (Figures 1(e) and 1(f)). 

**Figure 3:**
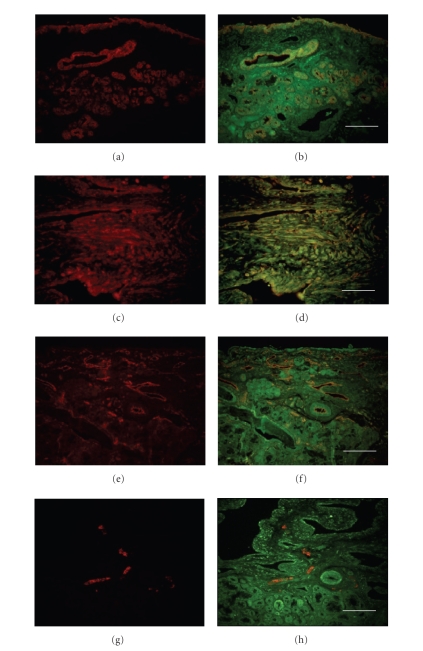
Identification of subsets of cells expressing the B1 receptor in human allergic nasal mucosa. Single staining immunofluorescence for each cell type (panel (a), (c), (e), and (g) and the dual staining for the cell marker and B1 receptor (panel (b), (d), (f), and (h). The B1 receptor protein (green) shows colocalization with antiphenotypical marker antibody (red) and the combined signal is visualized as yellow. Identification markers for cytokeratin (epithelial cells) (a), (b); fibroblast (c), (d); CD31 (vascular endothelial cells) (e), (f); neurofilament protein (peripheral nerves) (g), (h). Scale bar = 100 *μ*m.

**Figure 4:**
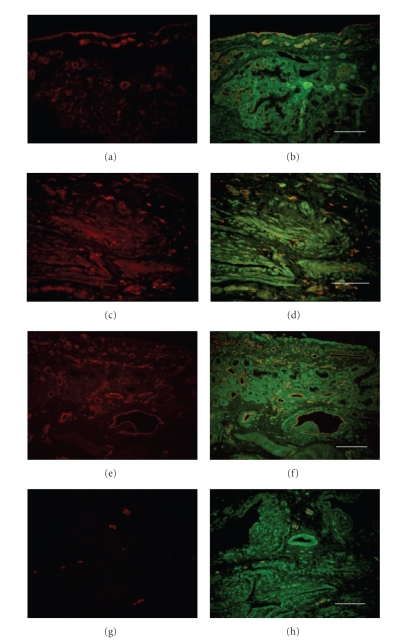
Identification of subsets of cells expressing the B2 receptor in human allergic nasal mucosa. Single staining immunofluorescence for each cell type (panel (a), (c), (e), and (g) and the dual staining for the cell marker and B2 receptor (panel (b), (d), (f), and (h). The B2 receptor protein (green) shows colocalization with antiphenotypical marker antibody (red) and the combined signal is visualized as yellow. Identification markers for cytokeratin (epithelial cells) (a), (b); fibroblast (c), (d); CD31 (vascular endothelial cells) (e), (f); neurofilament protein (peripheral nerves) (g), (h). Scale bar = 100 *μ*m.

**Figure 5:**
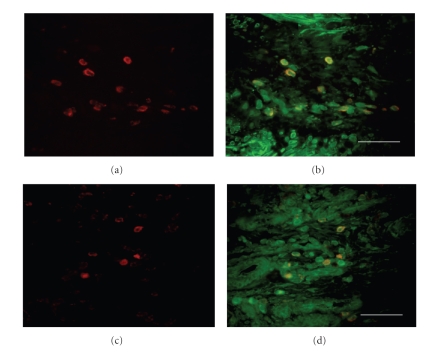
Expression of bradykinin B1 (a), (b) and B2 (c), (d) receptors on macrophages in human allergic nasal mucosa. (a) Macrophages (CD68-positive cells) (red). (b) Overlay image of bradykinin B1 receptor protein (green) and macrophages (CD68-positive) (red). The combined signal is visualized as yellow; (c) macrophages (CD68-positive) (red); (d) Overlay image of bradykinin B2 receptor protein (green); macrophages (CD68-positive cells) (red). Scale bar = 50 *μ*m.

In order to clarify the cell expressing bradykinin B1 and B2 receptors, we performed double immunofluorescence staining. As shown in Figures 3 and 4, epithelial cells, submucosal glands (Figures 3(b) and 4(b)), fibroblast (Figures 3(d) and 4(d)), vascular smooth muscle, and vascular endothelial cells (Figures 3(f) and 4(f)) express both B1 and B2 receptors. The B2 receptor was found in nerve fibers (Figure 4(h)), whereas the B1 expression was not observed in nerves (Figure 3(h)). As shown in Figure 5, the majority of CD68 positive macrophages showed immunoreactivity for both B1 and B2 receptors. 

The expression levels of both B1 and B2 receptors on epithelial cells and fibroblast were higher in allergic nasal mucosa (B1 receptor: Figures 1(a) and 1(c); B2 receptor: Figures 2(a) and 2(c)) than nonallergic nasal mucosa (B1 receptor: Figures 1(b) and 1(d); B2 receptor: Figures 2(b) and 2(d)). 

The patterns of the other immunohistochemical findings in all 12 cases were remarkably similar, and we could not find any other differences of B1 and B2 receptors immunoreactivity between on allergic and nonallergic nasal mucosae. The summary of the results is shown in Table 2. 

## 4. Discussion

It is well known that the responses to vasoactive kinin peptides are mediated through the activation of two receptors termed B1 and B2, which have been defined on the basis of the structure-function relationships of their agonists and antagonists [7]. The natural agonists of the B2-receptor are the nonapeptide bradykinin (BK) and the decapeptide Lys-BK (kallidin) which are generated by the proteolytic action of the serine protease kallikrein from the protein precursor kininogen [8]. BK and Lys-BK are weak B1 receptor agonist, however, the cleavage of these two B2-agonists by arginine carboxypeptidases produces the high affinity B1 receptor agonists, [des-Arg9]-BK, and [des-Arg10]-kallidin (DLBK), respectively, [7]. The B1 receptor is not expressed at significant levels in normal tissues, but its synthesis can be induced after tissue injury and by inflammatory factor such as lipopolysaccharide and IL-1 beta [9]. On the other hand, the B2 receptor is constitutively expressed in many types of the cells including smooth muscle cells, certain neurons, fibroblasts, and epithelial cells of the lung. 

In the present study, we confirm the expression of both bradykinin B1 and B2 receptors in human nasal epithelial cells. BK induced rise in [Ca^2+^] in primary cultured human nasal epithelial cells, suggesting the existence of B2 receptor on human nasal epithelial cells [10]. B1 receptor ligand, Lys-des-Arg-BK activated extracellular signal-regulated kinase (ERK) and the transcription factor AP-1 in human airway epithelial cell lines A549 and BEAS-2B [11]. Taken together, these previous observations and our present observations suggest the functional B1 and B2 receptors in human nasal epithelial cells. 

The present study indicated the significant expression of B1 and B2 receptor expressions on nasal fibroblasts. It has been reported that BK stimulated IL-1 [12], IL-6 [13], IL-8 [13, 14], and eotaxin [15] production in cultured human fibroblasts by increasing its gene expression. TNF-alpha and IL-1beta both induced an increase in B1 and B2 receptor expressions in human lung fibroblasts [16]. The observation of local production of IL-1*β* during inflammation accompanied by B1-receptor upregulation in several tissues has resulted in the hypothesis that this cytokine is directly involved in B1-receptor upregulation [9]. 

The expression of bradykinin B1 and B2 receptors were found not only on epithelial cells and fibroblasts, but also on submucosal glands in the present study. It has been reported that bradykinin receptors were detected over submucosal gland in human and guinea pig airways by in vitro autoradiography [17]. With respect to the effect of BK on airway submucosal glands, it has been reported that BK directly stimulates isolated airway submucosal gland cells and induces mucus glycoprotein and Cl^−^ secretion through the activation of B2 receptor [18]. Also, BK induces an increase in short-circuit current across a cultured gland cell layer from human airways with [Ca^2+^] rise, indicating a direct stimulation of ion transport in airway gland cells by BK [19]. On the other hand, some investigators have reported that BK had no significant effect on mucin release from human, feline, or ferret airway explants [6, 20]. 

In contrast to significant both B1 and B2 receptor expressions on epithelial cells, fibroblasts, and submucosal glands, B2 receptor expression on peripheral nerves, but not B1 receptor expression, could be detected by immunohistochemistry. B1 receptor was thought to be generally absent in healthy tissues [21, 22]. In contrast, B2 receptors are constitutively expressed in a range of cell types including sensory neurons, and their activation results in excitation and sensitization of sensory neurons [23, 24]. In B2 receptor agonist, BK can stimulate sensory nerve ending, causing the release of substance P and other neuropeptides [25]. It has been shown that the nasal stimulation with BK causes nasal pain [2, 3], suggesting the existence of the functional B2 receptor on sensory nerves. 

Using double immunofluorescence technique, we could confirm the significant expression of both B1 and B2 receptors on macrophages. The potency of kinins to stimulate leukocytes has been thought dependent on differentiation and especially on the activation stage of these cells. The differentiation of monocytes into macrophages is associated with functional and phenotypic changes. It has been shown that human peripheral monocytes express a low number of kinin B2 binding sites [26]. However, immature, unstimulated human monocytes-derived dendritic cells constitutively express both B1 and B2 receptors, whereas monocytes did not express B1 or B2 receptor protein [27], suggesting upregulation of B1 and B2 receptors during the differentiation of the cells. With respect to the effect of BK on monocytes, BK acting via B2 receptor, increased intracelluar Ca^2+^, and stimulated the migration of immature human monocyte-derived dendritic cells [27]. Thus, it might be possible that local macrophages might be activated by locally released BK during nasal allergic response. 

## 5. Conclusions

Using immunohistochemical technique, we have demonstrated the distribution of bradykinin B1 and B2 receptors in human nasal mucosa. Although kinins do not appear to have major role in allergic rhinitis, our findings should be of considerable interest for understanding the role of kinins on upper airway diseases such as allergic rhinitis and nonallergic rhinitis. 

## Figures and Tables

**Table 1:** Demographic characteristics of allergic and nonallergic patients.

	Allergic rhinitis	Nonallergic rhinitis
	*N* = 6	*N* = 6
Sex (male/female)	2/4	3/3
Age	31(19–58)	39(28–55)
Specific IgE to house dust mite (d1) (kU/L)	2.7(1.0–13)	<0.35
Total IgE (kU/L)	210(10–387)	110(10–185)
Blood eosinophils (cells/*μ*L)	370(70–690)	135(55–240)
Current nasal symptoms (number of patients)		
* *Nasal obstruction	6(all patients)	4(all patients)
* *Sneezing	4	0
* *Rhinorrhea	3	2

Data expressed as median values and range (in brankets).

**Table 2:** Distribution pattern of B1 and B2 receptors in normal and allergic nasal mucosae.

	Normal nasal		Allergic nasal	
	mucosa		mucosa	
	B1	B2	B1	B2
Epithelium	+	+	++	++
Submucosal gland	+	+	+	+
Nerve	−	++	−	++
Fibroblast	+	+	++	++
Vascular endothelial cell	+	+	+	+
Vascular smooth muscle	+	++	+	++
Inflammatory cell (macrophage)	++	+	++	+

## References

[1] Menke JG, Borkowski JA, Bierilo KK (1994). Expression cloning of a human B_1_ bradykinin receptor. *The Journal of Biological Chemistry*.

[2] Proud D, Reynolds CJ, Lacapra S, Kagey-Sobotka A, Lichtenstein LM, Naclerio RM (1988). Nasal provocation with bradykinin induces symptoms of rhinitis and a sore throat. *American Review of Respiratory Disease*.

[3] Austin CE, Foreman JC (1994). A study of the action of bradykinin and bradykinin analogues in the human nasal airway. *Journal of Physiology*.

[4] Dear JW, Wirth K, Scadding GK, Foreman JC (1996). Characterization of the bradykinin receptor in the human nasal airway using the binding of [125I]-Hoe 140. *British Journal of Pharmacology*.

[5] Austin CE, Foreman JC, Scadding GK (1994). Reduction by Hoe 140, the B_2_ kinin receptor antagonist, of antigen-induced nasal blockage. *British Journal of Pharmacology*.

[6] Baraniuk JN, Lundgren JD, Mizoguchi H (1990). Bradykinin and respiratory mucous membranes. Analysis of bradykinin binding site distribution and secretory responses in vitro and in vivo. *American Review of Respiratory Disease*.

[7] Regoli D, Barabé J (1980). Pharmacology of bradykinin and related kinins. *Pharmacological Reviews*.

[8] Bhoola KD, Figueroa CD, Worthy K (1992). Bioregulation of kinins: kallikreins, kininogens, and kininases. *Pharmacological Reviews*.

[9] Marceau F (1995). Kinin B_1_ receptors: a review. *Immunopharmacology*.

[10] Paradiso AM, Cheng EHC, Boucher RC (1991). Effects of bradykinin on intracellular calcium regulation in human ciliated airway epithelium. *American Journal of Physiology*.

[11] Christiansen SC, Eddleston J, Woessner KM (2002). Up-regulation of functional kinin B_1_ receptors in allergic airway inflammation. *The Journal of Immunology*.

[12] Pan ZK, Zuraw BL, Lung C-C, Prossnitz ER, Browning DD, Ye RD (1996). Bradykinin stimulates NF-*κ*B activation and interleukin 1*β* gene expression in cultured human fibroblasts. *The Journal of Clinical Investigation*.

[13] Hayashi R, Yamashita N, Matsui S (2000). Bradykinin stimulates IL-6 and IL-8 production by human lung fibroblasts through ERK- and p38 MAPK-dependent mechanisms. *European Respiratory Journal*.

[14] Hayashi R, Yamashita N, Matsui S (1998). Bradykinin stimulates interleukin-8 production by human lung fibroblasts. *Immunology*.

[15] Sato E, Nelson DK, Koyama S, Hoyt JC, Robbins RA (2000). Bradykinin stimulates eotaxin production by a human lung fibroblast cell line. *Journal of Allergy and Clinical Immunology*.

[16] Haddad E-B, Fox AJ, Rousell J (2000). Post-transcriptional regulation of bradykinin B1 and B2 receptor gene expression in human lung fibroblasts by tumor necrosis factor-*α*: modulation by dexamethasone. *Molecular Pharmacology*.

[17] Mak JCW, Barnes PJ (1991). Autoradiographic visualization of bradykinin receptors in human and guinea pig lung. *European Journal of Pharmacology*.

[18] Nagaki M, Shimura S, Irokawa T (1996). Bradykinin regulation of airway submucosal gland secretion: role of bradykinin receptor subtype. *American Journal of Physiology*.

[19] Yamaya M, Ohrui T, Finkbeiner WE, Widdicombe JH (1993). Calcium-dependent chloride secretion across cultures of human tracheal surface epithelium and glands. *American Journal of Physiology*.

[20] Sturgess J, Reid L (1972). An organ culture study of the effect of drugs on the secretory activity of the human bronchial submucosal gland. *Clinical Science*.

[21] Marceau F, Bachvarov DR (1998). Kinin receptors. *Clinical Reviews in Allergy and Immunology*.

[22] Walker K, Perkins M, Dray A (1995). Kinins and kinin receptors in the nervous system. *Neurochemistry International*.

[23] Banik RK, Kozaki Y, Sato J, Gera L, Mizumura K (2001). B_2_ receptor-mediated enhanced bradykinin sensitivity of rat cutaneous C-fiber nociceptors during persistent inflammation. *Journal of Neurophysiology*.

[24] Jeftinija S (1994). Bradykinin excites tetrodotoxin-resistant primary afferent fibers. *Brain Research*.

[25] Bertrand C, Geppetti P (1996). Tachykinin and kinin receptor antagonists: therapeutic perspectives in allergic airway disease. *Trends in Pharmacological Sciences*.

[26] Rajasekariah P, Warlow RS, Walls RS (1997). High affinity bradykinin binding to human inflammatory cells. *IUBMB Life*.

[27] Bertram CM, Baltic S, Misso NL (2007). Expression of kinin B_1_ and B_2_ receptors in immature, monocyte-derived dendritic cells and bradykinin-mediated increase in intracellular Ca2^+^ and cell migration. *Journal of Leukocyte Biology*.

